# A288 ALCOHOL RELAPSE AND ADVERSE OUTCOMES IN EARLY VERSUS ROUTINE LIVER TRANSPLANT FOR ALCOHOL-ASSOCIATED LIVER DISEASE IN THE PROVINCE OF BRITISH COLUMBIA

**DOI:** 10.1093/jcag/gwad061.288

**Published:** 2024-02-14

**Authors:** A R Hemy, A Fetz, S Jayakumar

**Affiliations:** The University of British Columbia, Vancouver, BC, Canada; The University of British Columbia, Vancouver, BC, Canada; The University of British Columbia, Vancouver, BC, Canada

## Abstract

**Background:**

Liver transplant is a life-saving treatment for patients with alcohol-associated liver disease (ALD) resulting in decompensated cirrhosis or severe alcoholic hepatitis, significantly reducing mortality compared to supportive treatment. Despite its previous wide acceptance, there is a lack of strong evidence to justify a 6-month abstinence period. In 2019, BC Transplant policies were updated to allow liver transplant within 6-months of alcohol use in carefully selected patients with limited life expectancy and favorable multidisciplinary psychosocial assessment.

**Aims:**

This study aims to assess alcohol relapse rates and adverse outcomes in patients who received an early liver transplant for ALD.

**Methods:**

A retrospective chart review was performed on all adult patients who underwent liver transplant in the province of British Columbia between January 1, 2020, and December 31, 2022. Follow up data was extracted until May 31, 2023. Patients were included if they had alcohol documented as a contributing factor to their liver disease prior to transplant. Early transplant was defined as alcohol abstinence of 179-days or less and routine transplant as 180-days or more. Alcohol use and time to alcohol use were determined by patient history, random biochemical testing, and health-care utilization for an alcohol-associated complication. Graft dysfunction or rejection resulting in a change in therapeutic management, noncompliance determined by clinician documentation, rehospitalization, and death were recorded as adverse events.

**Results:**

278 patients underwent liver transplant during the study period. 81 patients were classified as alcohol-related, and 15 received early transplant. The mean follow-up period was 20.5 months. Early transplant recipients were more likely to be younger (median 45 vs. 58 years, p = 0.003) and have a higher MELD score at the time of transplant (median 37 vs. 17, p ampersand:003C 0.001). There was no significant difference in post-transplant alcohol relapse (13 vs. 15%, hazard ratio = 0.78, 95% CI [0.17, 3.55], p = 0.74). Graft dysfunction was increased in patients who received early transplant (53 vs. 25%, relative risk = 2.17, 95% CI [1.15, 4.10]). There was no significant difference between rates of noncompliance (0 vs. 9%), rehospitalization (53 vs. 56%), or death (0 vs. 2%).

**Conclusions:**

Alcohol relapse is similar between early and routine liver transplant. Graft dysfunction is increased in early transplant, although other adverse events including medication noncompliance, rehospitalization, and death are not significant different. These results are favorable for the continued use of early liver transplant for ALD, providing an effective treatment for ALD and significantly reducing mortality in patients with a limited life expectancy.

**Funding Agencies:**

None

